# Genome-Wide Identification and Expression Profile of the SNAT Gene Family in Tobacco (*Nicotiana tabacum*)

**DOI:** 10.3389/fgene.2020.591984

**Published:** 2020-10-27

**Authors:** Jiemei Zhang, Zhengping Yao, Renjun Zhang, Zongmin Mou, Honghui Yin, Tianyang Xu, Dake Zhao, Suiyun Chen

**Affiliations:** ^1^Biocontrol Engineering Research Center of Plant Disease and Pest, Biocontrol Engineering Research Center of Crop Disease and Pest, Yunnan University, Kunming, China; ^2^School of Life Sciences, Yunnan University, Kunming, China; ^3^School of Ecology and Environmental Science, Yunnan University, Kunming, China; ^4^Wenshan Branch of Yunnan Tobacco Company, Wenshan, China

**Keywords:** *Nicotiana tabacum*, melatonin, serotonin *N*-acetyltransferase, tissue-specific expression, abiotic stress

## Abstract

Melatonin plays key roles in development and confers stress tolerance to plants. Serotonin *N*-acetyltransferase (SNAT) is either the enzyme involved in the last step or the penultimate enzyme of phytomelatonin biosynthesis. To date, *SNAT* genes have not been characterized in tobacco (*Nicotiana tabacum*), an economically important plant species. The sequence of the Acetyltransf_7 conserved domain was used as a query sequence, and 12 *NtSNAT* candidate genes were in turn identified in the genome of tobacco. These *NtSNAT*s could be divided into two groups based on the phylogenetic tree. *NtSNAT1* and *NtSNAT2* clustered together with the other typical *SNAT*s, but the other 10 *NtSNAT*s separately clustered outside of the typical *SNAT*s. These 10 *NtSNAT*s have only motif 1, whereas representative *SNAT*s, such as *NtSNAT1* and *NtSNAT2* or a *SNAT* from cyanobacteria, have five motifs. In addition, *NtSNAT1* and *NtSNAT2* are highly homologous to the characterized *OsSNAT1*, 62.95 and 71.36%, respectively; however, the homology between the other 10 *NtSNAT* genes and *OsSNAT1* is low. Concomitantly, it is hypothesized that *NtSNAT1* and *NtSNAT2* are the homolog of *SNATs*, whereas the other 10 candidates could be considered *NtSNAT*-like genes. Furthermore, both *Nicotiana tomentosiformis* and *Nicotiana sylvestris*, two diploid ancestor species of *N. tabacum*, have two *SNAT* candidates; therefore, it is speculated that gene rearrangement or deletion during the process of genomic stabilization after whole-genome duplication or polyploidization led to the preservation of *NtSNAT1* and *NtSNAT2* during the evolution of tobacco from the ancestral diploid to the allotetraploid. *NtSNAT* and *NtSNAT*-like genes were differentially expressed in all organs under different stress conditions, indicating that these genes potentially associated with plant growth and development and stress resistance. Under different stress conditions, the expression of *NtSNAT1* was significantly upregulated upon high-temperature and cadmium stresses, while the expression of *NtSNAT2* did not significantly increase under any of the tested stress treatments. These results provide valuable information for elucidating the evolutionary relationship of *SNAT* genes in tobacco and genetic resources for improving tobacco production in the future.

## Introduction

Melatonin (*N*-acetyl-5-methoxytryptamine) is an evolutionarily conserved pleiotropic molecule that exists ubiquitously in living organisms (Tan et al., 2011; Lee et al., 2014a; Kanwar et al., 2018). Melatonin is considered to be a growth regulator and is involved in regulating numerous plant biological processes, including seed germination, rooting, flowering, senescence, and photosynthesis (Chen et al., 2009; Kolář et al., 2010; Wang et al., 2012; Hernández et al., 2015; Wei et al., 2015; Zhang et al., 2017; Arnao and Hernández-Ruiz, 2019; Turk and Genisel, 2019). In addition to its roles in plant development, melatonin is also involved in the tolerance of plants to a range of biotic and abiotic stresses, such as pathogen infection, drought, high temperature, cold, and salinity (Tal et al., 2011; Byeon et al., 2014; Lee et al., 2014a; Shi et al., 2015a; Zhang et al., 2015; Arnao and Hernández-Ruiz, 2017; Zhao D. et al., 2019).

Melatonin is synthesized from tryptophan through four distinct reaction steps. A total of six enzymes, tryptophan decarboxylase (TDC), TPH, tryptamine 5-hydroxylase (T5H), serotonin *N*-acetyltransferase (SNAT), acetylserotonin-*O*-methyltransferase (ASMT), and caffeic acid *O*-methyltransferase (COMT), participate in the synthesis of melatonin in plants, among which SNAT is either the penultimate enzyme or the last step enzyme involved in melatonin biosynthesis (Back et al., 2016; Hardeland, 2016; Wang et al., 2017; Yu et al., 2018). Since the first plant *SNAT* gene was identified and cloned in rice, homologous *SNAT* genes in other species, such as cyanobacteria, alga laver, *Arabidopsis thaliana*, grapevine and apple, also have been identified, found to be present at even higher frequencies than the frequency in rice, and enzymatically characterized (Byeon et al., 2013, 2015, 2016b; Wang et al., 2017; Yu et al., 2019).

*SNAT*s have been reported to play a regulatory role in maintaining the steady-state level of melatonin, and there is a relationship between *SNAT*s and the response to abiotic stress (Park et al., 2014). A growing body of evidence indicates that different plant SNATs have different thermophilic properties: a SNAT protein of cyanobacteria presented increased catalytic activity at 70°C, a SNAT protein of loblolly pine presented increased catalytic activity at 55°C, and a SNAT protein of apple presented increased catalytic activity at 35°C. The heat resistance of SNATs shows that it functions in the heat stress response (Byeon et al., 2013, 2016b; Kang et al., 2013; Park et al., 2014; Yu et al., 2019). Additionally, transgenic *Arabidopsis* ectopically expressing *MzSNAT5* presented elevated melatonin levels, thus resulting in enhanced drought tolerance (Wang et al., 2017). Low melatonin production by suppression of *SNAT*s in rice causes stunted seedling growth together with yield penalty, increased abiotic stress susceptibility, and increased coleoptile growth under anoxic conditions (Byeon and Back, 2016). In addition to these abiotic defense responses, inhibition of the *GhSNAT1* melatonin biosynthesis-relate genes reduced the resistance of cotton inoculated with pathogenic bacteria (Li et al., 2019). Hence, *SNAT*s play an imperative role in the plant response to biotic and abiotic stresses.

Tobacco (*Nicotiana tabacum*) is an allotetraploid (2n = 48) that originated from chromosome doubling after an intraspecific hybridization event between *Nicotiana tomentosiformis* (2n = 24) and *Nicotiana sylvestris* (2n = 24) (Lim et al., 2004). Tobacco is an essential commercial crop species within the Solanaceae family. Tobacco is cultivated in more than 120 countries contributes substantially to the economic development of countries worldwide (Wang and Bennetzen, 2015; Tong et al., 2019). However, tobacco is often affected by various biotic and abiotic stresses during its growth and development, such as drought, pathogens, cold, high temperature, and heavy metals (Cho and Hong, 2006; Liu et al., 2011; Ma et al., 2014). These stresses cause stunted growth, senescence, reduce yields, and even death (Debnath et al., 2019). A large number of studies have confirmed that melatonin plays a pivotal role in plant growth and development and the response to biotic or abiotic stress (Hernández-Ruiz et al., 2004; Shi et al., 2015a,b; Yu et al., 2018; Zhao L. et al., 2019). However, numerous important biological pathways and gene families, including SNAT family members responsible for melatonin biosynthesis, remain unexplored in tobacco due to the lack of fully annotated reference genomes (Yu et al., 2020).

To further explore the roles of *SNAT* genes in tobacco, we used tobacco genomic data and performed a genome-wide investigation of the *SNAT* gene family. The phylogenetic relationships, sequence features, gene structures, and protein motifs of these *NtSNAT* genes were analyzed. Moreover, the functional diversity of *SNAT*s in tobacco was studied through the analysis of tissue-specific expression patterns and stress responses. Our results provide a reference for the identification of tobacco *SNAT* gene function and facilitate further work on improving the stress resistance of tobacco.

## Materials and Methods

### Tobacco *SNAT* Gene Sequence Retrieval and Gene Identification

To identify the tobacco *SNAT* candidates, the hidden Markov model (HMM) profile of the *SNAT* conserved domain (Pfam13508) was used as a query to search the genomic databases of *N. tabacum* (tobacco), *N. tomentosiformis*, *N. sylvestris*, and *Rhodospirillum rubrum* by the Markov model-based HMMER program. *R. rubrum* is the first photosynthetic α-proteobacterium indicated to synthesize melatonin (Manchester et al., 1995). All the candidate sequences were analyzed via the PFAM^1^ and SMART^2^ databases (Zhao Y. et al., 2019), and proteins without a typical *SNAT* conserved domain were removed. Chloroplast transit signal peptides were identified via ChloroP^3^ (Byeon et al., 2016a). The molecular weight (MW) and isoelectric point (pI) of each protein sequence were calculated using the online tool ExPASY^4^ (Liu et al., 2017).

### Distribution of Conserved Domains and Analysis of *NtSNAT* Structure

Conserved domains within *NtSNAT* genes were identified via the NCBI database^5^. Exon-intron structures were determined according to the alignments of their transcribed sequences with corresponding genomic sequences, and a diagram was generated with the online Gene Structure Display Server^6^. All the conserved motifs of the SNATs and NtSNAT proteins were subsequently identified by the MEME program^7^.

### Sequence Alignment and Phylogenetic Tree Construction

The deduced protein sequences of the NtSNATs were aligned with the sequence of OsSNAT1 (AK059369) using ClustalX (2.0.9). We performed phylogenetic analyses based on the neighbor-joining method using MEGA 7.0 software. Except for these of the *SNAT*s identified in *Nicotiana*, the sequences of other putative *SNAT*s were retrieved from the NCBI database (Supplementary Table S1). Bootstrap analysis was performed using 1,000 resampling replications, and branch lengths were assigned through pairwise calculations of the genetic distances.

### Plant Growth Conditions and Stress Treatments

Seeds of tobacco (K326 cultivar) were germinated in growth media consisting of a mixture of vermiculite and humus (v:v = 1:2) in polystyrene, square dishes. The seedlings were grown in a greenhouse at a day/night temperature of 25/18°C, an air humidity of 50–60%, and a photoperiod consisting of 16 h light/8 h dark; the light intensity was 100 μmol/m^2^/s. For heat-stress treatment, a group of 6 week-old tobacco seedlings was treated at 45°C in a growth chamber for 3, 6, and 9 h. For cold stress treatment, the seedlings were placed in a 4°C incubator for durations of 3, 6, and 24 h. The drought stress treatment was stopped, after which the seedlings were watered for 1, 4, and 7 days. For cadmium treatment, the cadmium concentration was determined on the basis of 10 mg/kg mixed soil, and samples were taken at 1, 4, and 7 days. The photoperiod and humidity of all the stress treatments were the same as those of the growth conditions described above. The conditions of the control treatment were the same those during seedling growth. Each treatment involved three biological replicates, and all the samples were immediately frozen in liquid nitrogen and stored at −80°C until analysis.

### Isolation of Total RNA and Reverse Transcription

Total RNA was extracted from tobacco leaves, stems, flowers, fruits, and roots using an RNAprep Pure Plant Kit (TIANGEN). First-strand cDNA was synthesized via a cDNA synthesis kit. Gene-specific primers were designed against the genome sequence of tobacco present in the NCBI database. qRT-PCRs assay were performed in a 10.0 μl reaction volume using Super Real PreMix Plus (TIANGEN), and the actin gene was used as an internal control. All the primers used for qRT-PCR-based analysis are presented in Supplementary Table S2. Expression data were calculated using the 2^–Δ^ ^Δ^ ^CT^ method, and the actin gene was used as a reference for the expression analysis of the *NtSNAT* genes in tobacco. All the results were generated via six samples: three biological replicates and three technical replicates. The 2^–Δ^ ^Δ^ ^CT^ values were subsequently used to draw heatmaps via MeV 4.9 and GraphPad Prism 5 software.

### Statistical Analysis

All the data were statistically analyzed using SPSS 23.0 statistical software. To investigate the expression differences of *SNAT* genes in all the samples, the *T*-test was used. Single asterisk (^∗^) indicate significant differences, at *p* ≤ 0.05, and double asterisks (^∗∗^) indicate extremely significant differences, at *p* ≤ 0.01.

## Results

### Primary Identification of *NtSNAT* Genes in the Tobacco Genome

**FIGURE 1:**
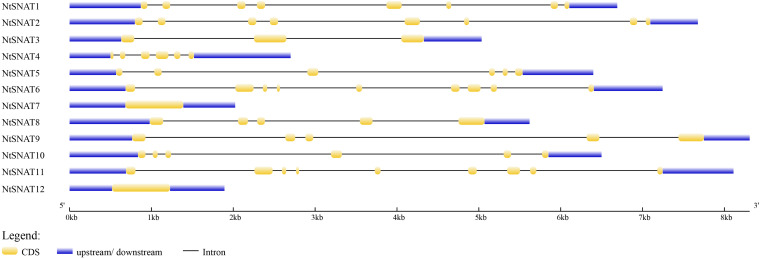
Exon-intron configurations of *NtSNAT* genes in tobacco plants. The introns and exons are drawn to scale together with the complete coding regions of their respective genes. The boxes indicate the exons, and lines indicate the introns.

Name searches and HMM analysis revealed a total of 12 candidate *SNAT* genes in tobacco. For convenience, we named the *NtSNAT* genes according to their MW. Details concerning the gene name, locus name, open reading frame (ORF) length, exon and intron numbers, protein length, MW, pI, and chloroplast transit signal peptide are listed in Table 1 and Supplementary Table S3. The MWs of the predicted NtSNAT proteins ranged from 20.4 to 34.9 kDa. In addition, the pIs ranged from 5.25 to 9.28, the ORF lengths ranged from 525 to 906, and protein lengths ranged from 174 to 301 amino acids (aa). A chloroplast transit signal peptide was identified in five of the candidate genes: *NtSNAT1*, *NtSNAT2*, *NtSNAT3*, *NtSNAT8*, and *NtSNAT9*. To examine the structural features of the *NtSNAT* genes, the exon/intron configurations of *NtSNAT* genes in the tobacco plants were compared. Sequence analysis revealed that introns were present in the coding DNA sequences (CDSs) of these genes, except in *NtSNAT7* and *NtSNAT12*, and the number of introns varied from 2 to 8 (Figure 1). On the basis of the established *SNAT* identification standard, for the two diploid ancestors of tobacco, *N. tomentosiformis*, and *N. sylvestris*, each have two *SNAT* genes.

**TABLE 1:** *SNAT* gene candidates in tobacco, *Nicotiana tomentosiformis*, and *Nicotiana sylvestris.*

**Name**	**Gene locus**	**ORF length (bp)**	**Chloroplast transit peptide**	**No.**	**No. introns**	**Deduced polypeptide**		
				**exons**		**Length (aa)**	**Mw (kDa)**	**pI**
*NtSNAT1*	LOC107779554	771	Yes	8	7	256	28.6	5.25
*NtSNAT2*	LOC107817186	768	Yes	8	7	255	28.4	5.38
*NtSNAT3*	LOC107796590	834	Yes	3	2	277	30.8	9.44
*NtSNAT4*	LOC107771832	651	No	6	5	216	25	8.95
*NtSNAT5*	LOC107772525	525	No	6	5	174	20.4	6.91
*NtSNAT6*	LOC107774960	906	No	9	8	301	34.9	9.23
*NtSNAT7*	LOC107791297	708	No	1	0	235	26.1	5.86
*NtSNAT8*	LOC107802269	855	Yes	5	4	284	31.7	7.53
*NtSNAT9*	LOC107820131	849	Yes	5	4	282	31.6	6.94
*NtSNAT10*	LOC107823851	525	No	6	5	174	20.4	6.91
*NtSNAT11*	LOC107827352	906	No	9	8	301	34.9	9.28
*NtSNAT12*	LOC107827499	708	No	1	0	235	26.3	5.68
*NsSNAT1*	LOC104229486	768	Yes	8	7	255	28.42	5.38
*NsSNAT2*	LOC104224393	780	Yes	1	0	259	21.91	9.8
*NtoSNAT1*	LOC104107124	768	Yes	8	7	255	28.59	5.15
*NtoSNAT2*	LOC104104574	780	Yes	1	0	259	21.86	9.58

### Multiple Sequence Alignment of *NtSNATs* and the Characterized Rice *OsSNAT1* Suggests *NtSNAT1* and *NtSNAT2* Are Authentic Tobacco *SNATs*

**FIGURE 2:**
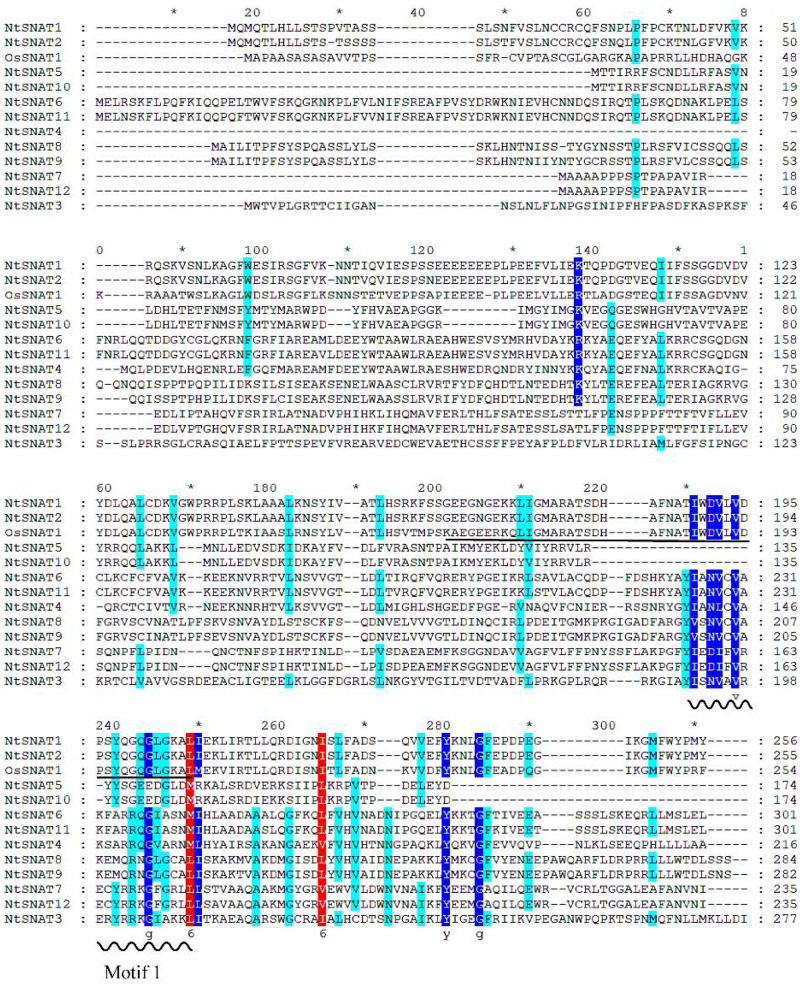
Multiple sequence alignment of OsSNAT1 and NtSNAT proteins. The wavy line in the figure indicates conserved motif 1, and the conserved acetyl-CoA binding motif is underlined. The conserved amino acid residues are indicated by colored shading. The asterisk “*” represent the length of every twenty amino acids (including blanks inside).

Sequence alignment of the NtSNAT proteins is shown in Figure 2. The results showed that *NtSNAT1* and *NtSNAT2* are highly homologous to the identified *OsSNAT1* (62.95 and 71.36%, respectively), while several other candidate genes have low homology with *OsSNAT1*. Therefore, we preliminarily infer that *NtSNAT1* and *NtSNAT2* are homologs of *SNATs* and that the other 10 members belong to another subgroup. It was found that aa 169–242 of *NtSNAT1* and *NtSNAT2* compose conserved domains according to NCBI conserved domain analysis. Each of these two proteins has a GNAT functional domain with a coenzyme-A-binding site, and it is suggested that these two proteins could be tobacco *SNAT*s. In addition, the motif 1 conserved region, which may have important functions, was detected in these proteins.

### Further Validation of the *NtSNAT1* and *NtSNAT2* Members of *NtSNAT* Family Based on Phylogenetic Tree and Motif Analysis

**FIGURE 3:**
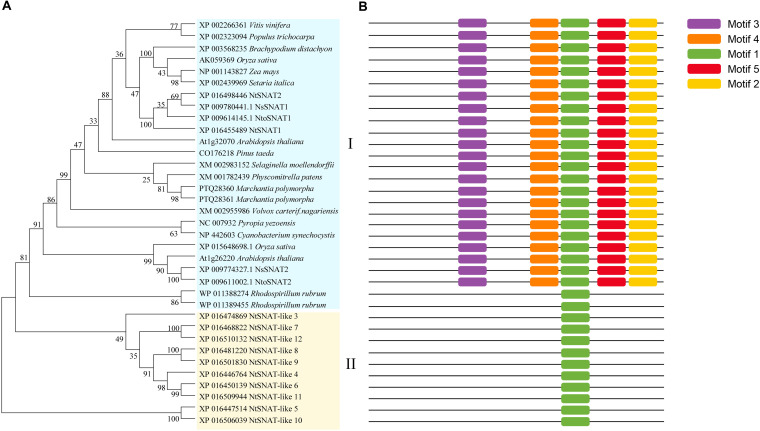
Phylogenetic tree of *NtSNAT*s and other *SNAT*s **(A)** and corresponding conserved motifs for each gene **(B)**. Different motifs are highlighted with different colored boxes with numbers 1–5.

For the phylogenetic tree, a BLAST search was performed using the rice *OsSNAT1* amino acid sequence. *SNAT* homologs were found in various taxa, including bacteria, algae, mosses, ferns, gymnosperms, and angiosperms (Figure 3A). These homologous genes not only have typical *SNAT* conserved domains but also complete conserved motifs. The phylogenetic tree indicated that the *NtSNAT* and *SNAT* genes from the included species could be divided into two clades. *NtSNAT1* and *NtSNAT2* could be classified as typical *SNAT*s, while the remaining 10 *NtSNAT*s clustered together separately. Additionally, four *SNAT*s from two diploid ancestors of tobacco, *N. tomentosiformis* and *N. sylvestris*, clustered into clade I together with the typical *SNAT*s. Protein motifs are often used to predict protein function. Twelve *NtSNAT* and twenty-three *SNAT* genes were analyzed according to their conserved motifs (Figure 3B and Supplementary Figure S1). The results showed that motif 1 was the most widely distributed and was present in all the members. However, except in *R. rubrum*, motif 2, motif 3, motif 4, and motif 5 were present in all the members of clade I. The *SNAT* genes in clade II contained only motif 1. Therefore, it was further speculated that *NtSNAT1* and *NtSNAT2* were likely tobacco *SNAT*s, and the other 10 candidates were considered *NtSNAT*-like genes that belonged to another subgroup. Each of the two ancestor diploids has two possible *SNAT* homologs. During the evolution from diploids to allotetraploids, there should be eight *NtSNAT* homologs in allotetraploid tobacco under normal circumstances, but only two *SNAT* homologs were identified in tobacco. These results suggest that the current typical *SNAT*s in tobacco may have arisen via gene loss during the process of genomic stabilization after the occurrence of polyploidization or whole-genome duplication. Moreover, motif 1, motif 2, motif 3, and motif 4 were present at the C-terminus, suggesting important biological functions of the C-terminus for members of the *SNAT* gene family.

### Organ-Specific Expression Analysis of *NtSNAT* and *NtSNAT*-Like Genes

**FIGURE 4:**
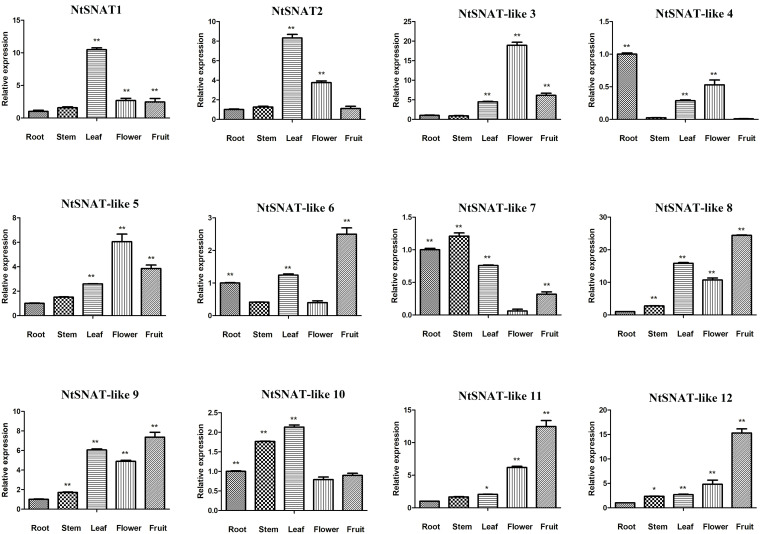
Expression profile of *NtSNAT* candidates in different tobacco organs based on qRT-PCR results. Single asterisk (*) indicates significant differences at *p* ≤ 0.05, and double asterisks (**) indicate extremely significant differences at *p* ≤ 0.01.

Expression profiling provides useful clues about gene function. To examine the expression patterns of the candidate *NtSNAT* genes, we evaluated their expression levels in the roots, stems, young leaves, flowers, and fruits (Figure 4). The results revealed that *NtSNAT* and *NtSNAT-*like genes were expressed in all of the tested organs. Among them, the expression of *NtSNAT1*, *NtSNAT2*, and *NtSNAT-*like 10 in the leaves was significantly higher than that in other organs. In addition, *NtSNAT-*like 3 and *NtSNAT-*like 5 were expressed at relatively high levels in the flowers. Similarly, *NtSNAT-*like 6, *NtSNAT-*like 8, *NtSNAT-*like 9, *NtSNAT-*like 11, and *NtSNAT-*like 12 were highly expressed in the fruits. The expression of *NtSNAT-*like 4 was extremely low in the stems and fruits, and this gene was expressed mainly in the roots. Last, the expression of *NtSNAT-*like 7 was significantly higher in the stems than in the other organs. And these genes related Gene Ontology terms were listed in Supplementary Table S4, further indicating the functions of 12 SNAT genes in different organs of tobacco.

### Expression Profiles of *NtSNAT* and *NtSNAT*-Like Genes Under Different Stress Conditions

**FIGURE 5:**
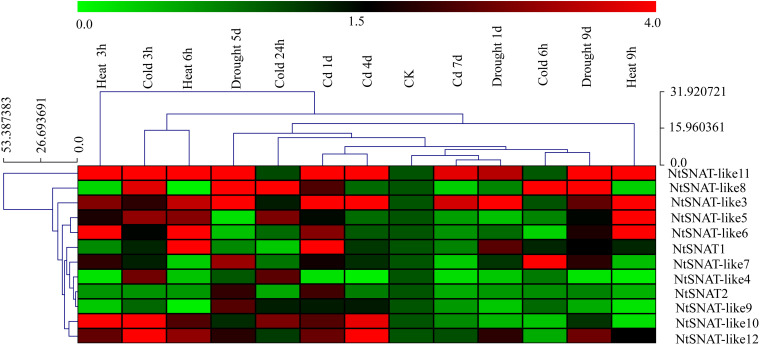
Hierarchical cluster display of expression profiles of *NtSNAT* and *NtSNAT*-like genes in tobacco leaves in response to drought, heat, cold, and cadmium (Cd) treatments. The blocks with colors indicate low (green) or high (red) transcript accumulation.

Under various stress conditions, it is evident that the expression of *NtSNAT* and *NtSNAT*-like genes was induced to a greater extent when the plants were subjected to abiotic stress, including heat, cold, cadmium, and drought than when they were under no stress (Figure 5). Furthermore, the expression of most of these genes was highly variable. The expression of six *NtSNAT* genes, *NtSNAT1*, *NtSNAT-*like 3, *NtSNAT-*like 5, *NtSNAT-*like 6, *NtSNAT-*like 10, and *NtSNAT-*like 11, dramatically increased in response to high-temperature conditions. Under cadmium stress, the expression of the *NtSNAT1*, *NtSNAT*-like 3, *NtSNAT-*like 10, *NtSNAT-*like 11, and *NtSNAT-*like 12 genes strongly increased. When the plants were under drought stress, the expression of only three genes, *NtSNAT-*like 3, *NtSNAT-*like 8, and *NtSNAT-*like 9, was significantly upregulated, whereas the *NtSNAT-*like 7, *NtSNAT-*like 8, *NtSNAT-*like 10, *NtSNAT-*like 11, and *NtSNAT-*like 12 genes responded strongly to cold stress. Furthermore, the expression of three of these genes (*NtSNAT2*, *NtSNAT-*like 4, and *NtSNAT-*like 9) did not significantly increase under all the tested stresses.

## Discussion

The SNAT family belongs to the GNAT superfamily, and the members of the SNAT family play a critical role in regulating the accumulation of melatonin (Dyda et al., 2000; Lee et al., 2015). *SNAT* genes have been identified in various plant species (Byeon et al., 2016b; Yu et al., 2019). Twelve candidate *SNAT* genes in tobacco were recognized. When aligned with the defined *OsSNAT1* amino acid sequence (Kang et al., 2013), *NtSNAT1* and *NtSNAT2* were highly homologous to *OsSNAT1* (62.95 and 71.36%, respectively), while several other *NtSNAT* genes were found to have low homology with *OsSNAT1*. Hence, we preliminarily determined that *NtSNAT1* and *NtSNAT2* might be tobacco *SNAT*s. Additionally, among the 12 candidate *NtSNAT* genes, only *NtSNAT1* and *NtSNAT2* have five conserved motifs, which is consistent with the findings of typical *SNAT*s. Moreover, *NtSNAT1* and *NtSNAT2* clustered together with the classic *SNAT*s, while the rest of the *NtSNAT*s clustered separately from the identified *SNAT*s. Therefore, these findings further confirmed our speculation.

Previous studies have shown that plant *SNAT*s are vertically transferred to descendants through endosymbiosis (Kang et al., 2013; Lee et al., 2014b; Byeon et al., 2015; Wang et al., 2020). The evolution of *SNAT* genes from cyanobacteria to higher plants verified that *NtSNAT1* and *NtSNAT2* indeed evolved vertically through endosymbiosis. From bacteria to higher plants, SNAT proteins are ubiquitous, which means that SNAT proteins may have evolved in the early stages of biological history. Aside from cyanobacteria, we also identified *SNAT* homologous genes from the purple non-sulfur bacteria *R. rubrum*, which has been proven to be able to synthesize melatonin (Tilden et al., 1997; Tan et al., 2013). Interestingly, *R. rubrum* is one of the most ancestral species of living organisms and is the first photosynthetic α-proteobacterium shown to synthesize melatonin (Manchester et al., 1995), despite only motif 1 being present in the *SNAT*s of *R. rubrum*. These facts, when taken together with all the tested species of common *SNAT* genes containing conserved motif 1, suggest that motif 1 is probably the core factor of *SNAT* genes.

Whole-genome duplication or polyploidization is an important driver of adaptation and speciation in plants (Hovav et al., 2008; Liu et al., 2019). In the polyploid genome, there are a large number of duplicated genes and duplicated genes from different diploid ancestors. Due to rearrangement or deletion of these duplicated genes, there are approximately three different fates of a polyploid genome, namely, subfunctionalization, pseudogenization or functional diversification (Adams et al., 2003; Hovav et al., 2008; Sojli et al., 2020). Therefore, it is speculated that the evolution of tobacco from being diploid (the two ancestral species of which were *N. tomentosiformis* and *N. sylvestris*) to allotetraploid may have caused the rearrangement or deletion of different genes due to gene duplication. In the present investigation, each of the two ancestor diploids has two possible *SNAT*s; however, only two typical *SNAT*s, *NtSNAT1* and *NtSNAT2*, were identified in tobacco. These results suggest that the typical *SNAT*s currently in tobacco may have arisen by gene loss during the process of genomic stabilization following polyploidization or whole-genome duplication. Ten additional *NtSNAT*-like genes branching independently from the typical *SNAT*s were identified, and the proteins encoded by these genes had markedly fewer motifs than *NtSNAT1* and *NtSNAT2* did or the representative *SNAT* from cyanobacteria did. Therefore, motif 1 has been stable throughout the evolutionary process.

To explore the possible functional differences of *NtSNAT* and *NtSNAT-*like genes, their expression patterns in different tissues and under different stresses were determined. The results demonstrated different types of expression patterns among these genes. With respect to the expression in different organs, 2 *NtSNAT* and 10 *NtSNAT-*like genes were expressed in the roots, stems, leaves, flowers, and fruits, indicating that these genes may have potential effects in vegetative and reproductive growth. Moreover, the high expression of *NtSNAT1* and *NtSNAT2* in the leaves indicates that these two genes could be pivotal in leaf growth and development. In addition, studies have shown that *SNAT*s play an important role in the process of plant stress resistance (Wang et al., 2017). The SNAT enzyme is involved in the biosynthesis of melatonin, which is reported to regulate the thermotolerance of many plant species. For example, the cyanobacteria *SNAT* gene was proven to be involved in melatonin in response to high temperature (Byeon et al., 2013). Under heat-stress conditions, *SlSNAT* interacts with HSP40 to maintain melatonin levels, thereby increasing the heat resistance of tomato plants (Wang et al., 2020). In this study, *NtSNAT1*, *NtSNAT-*like 3, *NtSNAT-*like 5, *NtSNAT-*like 6, and *NtSNAT-*like 10 responded to heat stress at different heat treatment time points, indicating that these genes may have potential effects in increasing heat resistance. Similarly, Lee and Back (2017) showed that overexpression of *OsSNAT* in rice can significantly increase plant resistance to cadmium stress and senescence. In this paper, under cadmium-stress conditions, *NtSNAT1*, *NtSNAT-*like 3, *NtSNAT-*like 10, *NtSNAT-*like 11, and *NtSNAT-*like 12 were found to respond to cadmium stress at different time points, suggesting that these genes potentially associated with increasing plant tolerance to cadmium. Notably, the expression of *NtSNAT2*, a representative *SNAT* gene, was not significantly upregulated compared with that in the control group under all the stress treatments. Accordingly, it is speculated that *NtSNAT2* may not respond during the actual stress time and may be expressed at other time points. The exact role of *NtSNAT1* and *NtSNAT2* and the catalytic activities of their encoded proteins require further study.

## Conclusion

Serotonin *N*-acetyltransferase is a key enzyme in the melatonin biosynthesis pathway. *NtSNAT1* and *NtSNAT2*, together with 10 additional *NtSNAT*-like genes, were identified as candidate genes for improving tobacco production. Among the five motifs typically present within *SNAT*s, motif 1 is indispensable for melatonin biosynthesis. During the evolutionary process through which tobacco changed from being diploid to allotetraploid, *NtSNAT1* and *NtSNAT2* were retained due to gene rearrangement or deletion during genome stabilization after whole-genome duplication and polyploidization. *NtSNAT1* potentially associated with regulating plant growth and development and increasing plant tolerance to stress.

## Data Availability Statement

All datasets presented in this study are included in the article/Supplementary Material.

## Author Contributions

DZ and SC designed the experiments. JZ designed and carried out the experiments and wrote the manuscript. ZY, RZ, and ZM participated and analyzed the data from the experiments. All the authors reviewed and approved the manuscript in its final form.

## Conflict of Interest

HY and TX was employed by the Wenshan Branch of Yunnan Tobacco Company. The remaining authors declare that the research was conducted in the absence of any commercial or financial relationships that could be construed as a potential conflict of interest.

## Footnotes

1 http://pfam.xfam.org/

2 http://smart.embl-heidelberg.de/

3 http://www.cbs.dtu.dk/services/Chlorop/

4 http://web.expas-y.org/compute_pi

5 https://www.ncbi.nlm.nih.gov/Strucure/cd-d/Wrpsb.cgi

6 http://www.cbi.pku.edu.cn/resource/index.htm

7 http://meme-suite.org/tools/meme

## Supplementary Material

Supplementary Figure 1 Conserved amino acid sequences of 12 motifs.

Supplementary Table 1 Accession number and source organism of 35 SNATs from bacteria, algae, mosses, ferns, gymnosperms, and angiosperms.

Supplementary Table 2 Primers used for the qRT-PCR assay of 12 candidate *NtSNAT*s.

Supplementary Table 3 Full amino acid sequences of 12 candidate *NtSNAT*s.

Supplementary Table 4 *NtSNAT* and *NtSNAT*-like genes and their related Gene Ontology (GO) terms.
