# Supplementary material for: Genome-Wide Identification and Expression Profile of the SNAT Gene Family in Tobacco (Nicotiana tabacum)
Source: Front Genet. 2020 Oct 27;11:591984. doi: 10.3389/fgene.2020.591984 (PMC7652900; doi:10.3389/fgene.2020.591984)
Supplement: Supplementary Figure 1 — Conserved amino acid sequences of 12 motifs. [file Data_Sheet_1.docx]

Supplementary Material

**Table S1. The accession number and source organnisms of the 35 SNAT that was used to construct the phylogenetic tree, SNAT proteins from bacteria, algae, mosses, ferns, gymnosperms and angiosperms**

| Accession number | Source organisms |
| --- | --- |
| AK659369 | *Oryza sativa* |
| XP_015648698.1 | *Oryza sativa* |
| At1g32070 | *Arabidopsis thaliana* |
| At1g26220 | *Arabidopsis thaliana* |
| NP_442603 | *Cyanobacterium synechocystis* |
| XM_002983152 | *Selaginella moellendorffii* |
| NP_001143827 | *Zea mays* |
| XP_003568235.1 | *Brachypodium distachyon* |
| XP_002266361.1 | *Vitis vinifera* |
| XP_002323094.1 | *Populus trichocarpa* |
| XM_001782439 | *Physcomitrella patens* |
| XM_002955986 | *Volvox carterif.nagariensis* |
| NC_007932  XP_002439969.1 | *Pyropia yezoensis*  *Setaria italica* |
| PTQ28360 | *Marchantia polymorpha* |
| PTQ28361 | *Marchantia polymorpha* |
| WP_011388274 | *Rhodospirillum rubrum* |
| WP_011389455  CO176218  XP_009780441.1  XP_009774327.1  XP_009614145.1  XP_009611002.1 | *Rhodospirillum rubrum*  *Pinus taeda*  *Nicotiana sylvestris*  *Nicotiana sylvestris*  *Nicotiana tomentosiformis*  *Nicotiana tomentosiformis* |
| XP_016455489.1 | *NtSNAT1* |
| XP_016498446.1 | *NtSNAT2* |
| XP_016474869.1 | *NtSNAT3* |
| XP_016446764.1 | *NtSNAT4* |
| XP_016447514.1 | *NtSNAT5* |
| XP_016450139.1 | *NtSNAT6* |
| XP_016468822.1 | *NtSNAT7* |
| XP_016481220.1 | *NtSNAT8* |
| XP_016501830.1 | *NtSNAT9* |
| XP_016506039.1 | *NtSNAT10* |
| XP_016509944.1 | *NtSNAT11* |
| XP_016510132.1 | *NtSNAT12* |

| **Primers Name** | **Primer sequence** |
| --- | --- |
| NtSNAT1-F  NtSNAT1-R  NtSNAT2-F  NtSNAT2-R  NtSNAT3-F  NtSNAT3-R  NtSNAT4-F  NtSNAT4-R  NtSNAT5-F  NtSNAT5-R  NtSNAT6-F  NtSNAT6-R  NtSNAT7-F  NtSNAT7-F  NtSNAT8-F  NtSNAT8-R  NtSNAT9-F  NtSNAT9-R  NtSNAT10-F  NtSNAT10-R  NtSNAT11-F  NtSNAT11-R  NtSNAT12-F  NtSNAT12-R  Actin-F  Actin-R | \| GACTCAACCAGATGGAACAGTCG \| \| --- \| \| GCTTCTTTTCTCCGTTCCCCT \| \| TCCGGGTTTGTTAAGAATAACACTG \| \| CTTCGAGGCCATCCAACCTT \| \| ATGTGACTGGAATATTGACCGTGGA \| \| TGTGCCTCCGCTTTAGTTATGA \| \| TCCTGGGGAAAGGGTGAATG \| \| CACGAACACCTTTTCTGCACC \| \| GAGCATCAAATACGCCAGCC \| \| GCCGCTTAAGGGGGATGATA \| \| AGGTTATCAGCTGTCTTGGCA \| \| TTGAAACCTTGTAAGGCGGC \| \| CTTAATTCCGACTGCCCACC \| \| GGTAAAGGTAGTGAAGGGAGGC \| \| CTCAACAACTTTCCCAACAAAACC \| \| CGATACGTTCCGTCAATGCT \| \| ATGGCAATCCTCATTACACCAT \| \| GGTGAAGAAATTTGTTGGGAAAGT \| \| GTGGACCTGTTTGTGAGAGC \| \| CAATTCATCCGGAGTTACTGGC \| \| TCGACGTCAGGGTATTGCTT \| \| AGAGACGACGAAGTTTCTTCCA \| \| CGACGTTCCCCATATCCACAA \| \| ACTTCGAGGAGGAAGATGGTG  AACTGGGACGATATGGAGAA  CACTGGCGTATAGGGACAAC \| |

**Table S2. Primers used in this paper.**

**Table S3. The full amino acid sequences of the twelve NtSNAT.**

**NtSNAT1**

MQMQTLHLLSTSPVTASSSLSNFVSLNCCRCQFSNPLPFPCKTNLDFVKVKRQSKVSNLKAGFWESIRSGFVKNNTIQVIESPSSEEEEEEEPLPEEFVLIEKTQPDGTVEQIIFSSGGDVDVYDLQALCDKVGWPRRPLSKLAAALKNSYIVATLHSRKFSSGEEGNGEKKLIGMARATSDHAFNATIWDVLVDPSYQGQGLGKALIEKLIRTLLQRDIGNISLFADSQVVEFYKNLGFEPDPEGIKGMFWYPMY

**NtSNAT2**

MQMQTLHLLSTSTSSSSSLSTFVSLNCCRCQFSNQLPFPCKTNLGFVKVKRQSKVSNLKAGFWESIRSGFVKNNTVQVIESPSNEEEEEEEPLPEEFVLIEKTQPDGTVEQIIFSSGGDVDVYDLQALCDKVGWPRRPLSKLAAALKNSYIVATLHSRKFSSGEEGNGEKKLIGMARATSDHAFNATIWDVLVDPSYQGQGLGKALIEKLIKTLLQRDIGNISLFADSQVVEFYKNLGFEPDPEGIKGMFWYPMY

**NtSNAT3**

MWTVPLGRTTCIIGANNSLNLFLNPGSINIPFHFPASDFKASPKSFSSLPRRSGLCRASQIAELFPTTSPEVFVREARVEDCWEVAETHCSSFFPEYAFPLDFVLRIDRLIAMLFGFSIPNGCKRTCLVAVVGSRDEEACLIGTEELKLGGFDGRLSLNKGYVTGILTVDTVADFLPRKGPLRQRRKGIAYISNVAVRERYRRKGIAKKLITKAEAQARSWGCRAIALHCDTSNPGAIKLYIGEGFRIIKVPEGANWPQPKTSPNMQFNLLMKLLDI

**NtSNAT4**

MQLPDEVLHQENRLEFGQFMAREAMFDEEYWTAAWLRAESHWEDRQNDRYINNYKKQYAEQEFNALKRRCKAQIGQRCTCIVTVRNEEKNNRHTVLKSVVGTLDLMIGHLSHGEDFPGERVNAQVFCNIERRSSNRYGYIANLCVAKSARRQGVARNMLHYAIRSAKANGAEKVFVHVHTNNGPAQKLYQKVGFEVVQVPNLKLSEEQPHLLLLAA

**NtSNAT5**

MTTIRRFSCNDLLRFASVNLDHLTETFNMSFYMTYMARWPDYFHVAEAPGGKIMGYIMGKVEGQGESWHGHVTAVTVAPEYRRQQLAKKLMNLLEDVSDKIDKAYFVDLFVRASNTPAIKMYEKLDYVIYRRVLRYYSGEEDGLDMRKALSRDVERKSIIPLKRPVTPDELEYD

**NtSNAT6**

MELRSKFLPQFKIQQPELTWVFSKQGKNKPLFVLNIFSREAFPVSYDRWKNIEVHCNNDQSIRQTPLSKQDNAKLPELSFNRLQQTDDGYCGLQKRNFGRFIAREAMLDEEYWTAAWLRAEAHWESVSYMRHVDAYKRKYAEQEFYALKRRCSGQDGNCLKCFCFVAVKKEEKNVRRTVLNSVVGTLDLTIRQFVQRERYPGEIKRLSAVLACQDPFDSHKYAYIANVCVAKFARRQGIASNMIHLAADAAALQGFKQLFVHVNADNIPGQELYKKTGFTIVEEASSSLSKEQRLLMSLEL

**NtSNAT7**

MAAAAPPPSPTPAPAVIREDLIPTAHQVFSRIRLATNADVPHIHKLIHQMAVFERLTHLFSATESSLSTTLFPENSPPPFTTFTVFLLEVSQNPFLPIDNQNCTNFSPIHKTINLDLPVSDAEAEMFKSGGNDAVVAGFVLFFPNYSSFLAKPGFYIEDIFVRECYRRKGFGRLLLSTVAAQAAKMGYGRVEWVVLDWNVNAIKFYEEMGAQILQEWRVCRLTGGALEAFANVNI

**NtSNAT8**

MAILITPFSYSPQASSLYLSSKLHNTNISSTYGYNSSTPLRSFVICSSQQLSQQNQQISPPTPQPILIDKSILSISEAKSENELWAASCLRVRTFYDFQHDTLNTEDHTKYLTEREFEALTERIAGKRVGFGRVSCVNATLPFSKVSNVAYDLSTSCKFSQDNVELVVVGTLDINQCIRLPDEITGMKPKGIGADFARGYVSNVCVAKEMQRNGLGCALISKAKMVAKDMGISDLYVHVAIDNEPAKKLYMKCGFVYENEEPAWQARFLDRPRRLLLWTDLSSS

**NtSNAT9**

MAILITPFSYSPQASSLYLSSKLHNTNIIYNTYGCRSSTPLRSFVLCSSQQLSQQISSPTPHPILIDKSFLCISEAKSENELWAASSLRVRIFYDFQHDTLNTEDHTKYLTEREFEALTERIAGKRVGFGRVSCINATLPFSEVSNVAYDLSTSCKFSQDNVELVVVGTLDINQCIRLPDEITGMKPKGIGADFARGYVSNVCVAKEMQRNGLGCALISKAKTVAKDMGISDLYVHVAIDNEPAKKLYMKCGFVYENEEPAWQARFLDRPRRLLLWTDLSNS

**NtSNAT10**

MTTIRRFSCNDLLRFASVNLDHLTETFNMSFYMTYMARWPDYFHVAEAPGGRIMGYIMGKVEGQGESWHGHVTAVTVAPEYRRQQLAKKLMNLLEDVSDKIDKAYFVDLFVRASNTPAIKMYEKLDYVIYRRVLRYYSGEEDGLDMRKALSRDIEKKSIIPLKRPVTPDELEYD

**NtSNAT11**

MELNSKFLPQFKIQQPQFTWVFSKQGKNKPLFVVNIFSREAFPVSYDRWKNIEVHCNNDQSIRQTPLSKQDNAKLPELSFNRLQQTDDGYCGLQKRNFGRFIAREAVLDEEYWTAAWLRAEAHWESVSYMRHVDAYKRKYAEQEFYALKRRCSGQDGNCLKCFCFVAVKKEEKNVRRTVLNSVVGTLDLTVRQFVQRERYPGEIKKLSTVLACQDPFDSHKYAYIANVCVAKFARRQGIASNMIHLAADAASLQGFKQLFVHVNADNIPGQELYKKTGFKIVEETSSSLSKEQRLLMSLEL

**NtSNAT12**

MAAAAPPPSPTPAPAVIREDLVPTGHQVFSRIRLATNADVPHIHKFIHQMAVFERLTHLFSATESSLSATLFPENSPPPFTTFTIFLLEVSQNPFLPIDNQNCTNFSPIHKTINLDLPISDPEAEMFKSGGNDEVVAGFVLFFPNYSSFLAKPGFYIEDIFVRECYRRKGFGRLLLSAVAAQAAKMGYGRVEWVVLDWNVNAIKFYEEMGAQILQEWRVCRLTGGALEAFANVNI

**NsSNAT1**

MQMQTLHLLSTSTSSSSSLSTFVSLNCCRCQFSNQLPFPCKTNLGFVKVKRQSKVSNLKAGFWESIRSGFVKNNTVQVIESPSNEEEEEEEPLPEEFVLIEKTQPDGTVEQIIFSSGGDVDVYDLQALCDKVGWPRRPLSKLAAALKNSYIVATLHSRKFSSGEEGNGEKKLIGMARATSDHAFNATIWDVLVDPSYQGQGLGKALIEKLIKTLLQRDIGNISLFADSQVVEFYKNLGFEPDPEGIKGMFWYPMY

**NsSNAT2** MLLYNPISTHLPPTPTALSLKPTTIHHRNVIVSSQYQPIPTTVNISISDESLKSKGFNLHRSITNLNLDH

LNSVFVAVGFPRRDTTKIQLALENTDSLMWIEYEKTKRPVAFARATGDGVFNAIIWDVVVDPNFQGIGLG

KAVMERLVTELLGKGINNIALYSEPRVLGFYRPLGFVADPDGIRGMVYSRKKKKNR

**NtoSNAT1**

MQMQTLYLLSTSPVTASSSLSNFVSLNCCRCQFSNPLPFPCKTNLDFVKVKRQSKVSNLKAGFWESIRSGFVKNNTIQVIESPSSEEEEEEEPLPEEFVLIEKTQPDGTVEQIIFSSGGDVDVYDLQALCDKVGWPRRPLSKLAAALKNSYIVATLHSRKFSSGEEGNGEKKLIGMARATSDHAFNATIWDVLVDPSYQGQGLGKALIEKLIRTLLQRDIGNISLFADSQVVEFYKNLGFEPDPEGIKGMFWYPMY

**NtoSNAT2**

MLLYNPISTHLPPTPLTLKPTTHHHHQNVTVSSQYQPIPTTVNISISDESLKSKGFNLHRSITNLNLDHLNSVFVAVGFPRRDTTKIQLALENTDSLAWIEYEKTKRPVAFARATGDGVFNAIIWDVVVDPNFQGIGLGKAVMERLVTELLEKGISNIALYSEPRVLGFYRPLGFVADPDGIRGMVYSRKKKKNR

**Table S4**. ***NtSNAT* and *NtSNAT-*like genes and their related Gene Ontology (GO) terms.**

|  | ID | Terms | Genes | Number  of genes |
| --- | --- | --- | --- | --- |
| Molecular Function | GO:0008080  GO:0016740 | *N*-acetyltransferase activity  transferase activity | *NtSNAT1, NtSNAT2, NtSNAT-*like*3, NtSNAT-*like*4, NtSNAT-*like*5, NtSNAT-*like*6, NtSNAT-*like*7, NtSNAT-*like*8, NtSNAT-*like*9, NtSNAT-*like*10,*  *NtSNAT-*like*11, NtSNAT-*like*12* | 12 |
|  | GO:0004596 | peptide alpha-*N*-acetyltransferase activity | *NtSNAT-*like*10* | 1 |
|  | GO:0016747 | Transferase activity, transferring acyl groups other than amino-acyl groups | *NtSNAT-*like*7, NtSNAT-*like*12* | 2 |
| Celluar Component | GO:0009507 | chloroplast | *NtSNAT-*like*8, NtSNAT-*like*9* | 2 |
|  | GO:0031416 | NatB complex | *NtSNAT-*like*10* | 1 |
| Biological Process | GO:0017196 | *N*-terminal peptidyl-methionine acetylation | *NtSNAT-*like*10* | 1 |


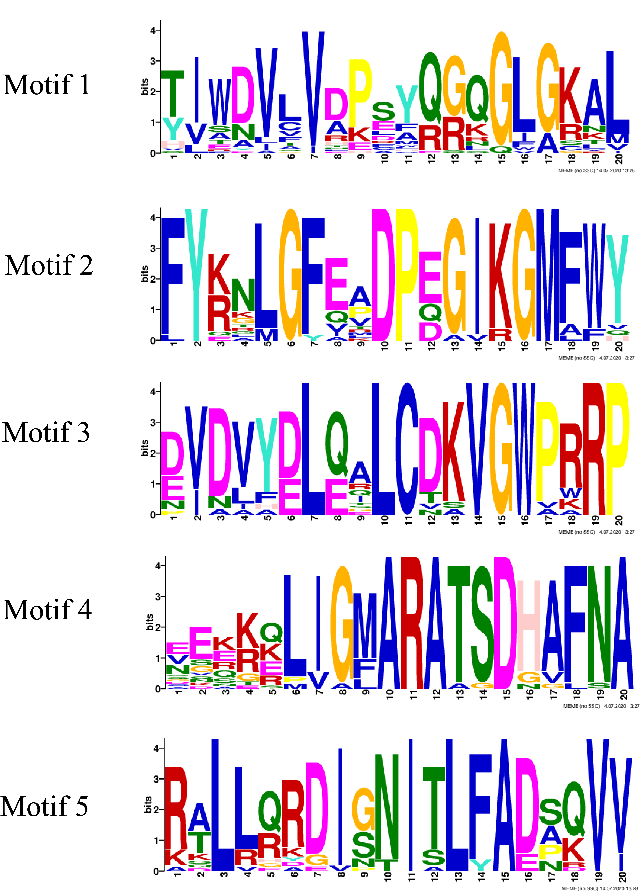


**Supplementary Figure 1. Conserved motif map**
